# Activated Macrophage Survival Is Coordinated by TAK1 Binding Proteins

**DOI:** 10.1371/journal.pone.0094982

**Published:** 2014-04-15

**Authors:** September R. Mihaly, Sho Morioka, Jun Ninomiya-Tsuji, Giichi Takaesu

**Affiliations:** 1 Department of Biological Sciences, Environmental and Molecular Toxicology, North Carolina State University, Raleigh, North Carolina, United States of America; 2 Center for Integrated Medical Research, Department of Microbiology and Immunology, Keio University School of Medicine, Tokyo, Japan; University of Illinois at Chicago, United States of America

## Abstract

Macrophages play diverse roles in tissue homeostasis and immunity, and canonically activated macrophages are critically associated with acute inflammatory responses. It is known that activated macrophages undergo cell death after transient activation in some settings, and the viability of macrophages impacts on inflammatory status. Here we report that TGFβ- activated kinase (TAK1) activators, TAK1-binding protein 1 (TAB1) and TAK1-binding protein 2 (TAB2), are critical molecules in the regulation of activated macrophage survival. While deletion of *Tak1* induced cell death in bone marrow derived macrophages even without activation, *Tab1* or *Tab2* deletion alone did not profoundly affect survival of naïve macrophages. However, in lipopolysaccharide (LPS)-activated macrophages, even single deletion of *Tab1* or *Tab2* resulted in macrophage death with both necrotic and apoptotic features. We show that TAB1 and TAB2 were redundantly involved in LPS-induced TAK1 activation in macrophages. These results demonstrate that TAK1 activity is the key to activated macrophage survival. Finally, in an *in vivo* setting, *Tab1* deficiency impaired increase of peritoneal macrophages upon LPS challenge, suggesting that TAK1 complex regulation of macrophages may participate in *in vivo* macrophage homeostasis. Our results demonstrate that TAB1 and TAB2 are required for activated macrophages, making TAB1 and TAB2 effective targets to control inflammation by modulating macrophage survival.

## Introduction

Macrophages are characterized by phagocytic activity, and play diverse roles in different tissue types. While resident macrophages participate in morphogenesis and tissue homeostasis, resident and recruited macrophages also play a major role in acute inflammatory responses [1]. Upon tissue injury or invasion by microorganisms, circulating inflammatory monocytes are recruited and differentiated toward mature macrophages. These macrophages are canonically activated by necrotic debris and bacterial moieties through Toll-like receptor signaling pathway, developing into so-called M1 polarized macrophages [2]. Activated macrophages clean dead cells and microorganisms by phagocytosis and produce inflammatory cytokines resulting in amplification of inflammation. Subsequently, these activated macrophages are deactivated or killed to terminate inflammatory conditions. In some experimental settings, it is known that lipopolysaccharide (LPS)-induced activation of macrophages reduces macrophage viability [3]–[5]. However, the mechanism by which activated macrophages undergo cell death is still largely elusive.

TGFβ- activated kinase (TAK1) is a member of the mitogen-activated protein kinase kinase kinase (MAPKKK) family, and is an indispensable intermediate of cytokine and Toll-like receptor pathways [6]–[8]. TAK1 is recruited to and activated by the receptor proximal complex of TNF, IL-1, and Toll-like receptors through a poly-ubiquitin chain-mediated mechanism [9]. TAK1-binding protein 2 (TAB2) and its closely related protein, TAK1-binding protein 3 (TAB3), have ubiquitin binding domains and tether between TAK1 and the poly-ubiquitin chain resulting in activation of TAK1 [10]–[14]. TAB2 and TAB3 may redundantly function in innate immune pathways, but TAB2 plays an indispensable role at least during development [15]. Additionally, it has recently been shown that *Tab3* deletion does not impair innate or adaptive immunity [16]. Thus, TAB2 is the major adaptor between TAK1 and activating poly-ubiquitin chains in immune cells. TAK1 is also activated through another binding partner, TAK1-binding protein 1 (TAB1), which is structurally unrelated to TAB2/3 and binds to TAK1 at a site different from the TAB2/3-binding site [17], [18]. TAB1 is found to be constantly associated with TAK1, and we recently demonstrated that TAB1 is involved in stress-dependent TAK1 activation [19] and activity of TAK1 in epithelial tissues [20]. Major known downstream molecules of TAK1 are IκB-kinases (IKKs) and mitogen-activated protein kinases (MAPKs) including p38 and JNK, which in turn activate transcription factors NF-κB and AP-1, respectively.

*In vivo*, TAK1 signaling is found to be important for immune responses in T and B cells through regulation of NF-κB and MAPK pathways in a mouse model [21]–[24], which is anticipated from the results in the tissue culture system described above. However, unexpectedly, the most overt phenotype caused by *Tak1* deficiency *in vivo* is tissue damage associated with cell death in the epidermis, intestinal epithelium and liver [25]–[31]. Since *Tak1* deficiency does not cause cell death in primary culture fibroblasts or keratinocytes, the cell death must be induced depending on the *in vivo* environment. TAK1 has been found to be integral to prevent tissue-derived TNF-induced cell death *in vivo*, which is evidenced by the fact that *Tnfr1* deletion can rescue cell death and tissue damage in these tissues [26], [27], [29]. Single deletion of *Tab1* or *Tab2* does not cause any abnormalities in the epidermis and intestinal epithelium but double deletion of *Tab1* and *Tab2* phenocopies *Tak1* deficiency [20], suggesting that TAB1 and TAB2 redundantly function in TAK1 regulation in these tissues. However, the specific roles of TAB1 and TAB2 in adult tissues are still largely elusive.

Recent studies have demonstrated that *Tak1* deficiency in myeloid cells results in hyper-proliferation of neutrophils and increased inflammatory conditions [32], [33]. Bone marrow derived macrophages (BMDMs) generated from myeloid-specific *Tak1*-deficient mice have been reported to undergo spontaneous cell death under normal culture conditions [32], [34]. These studies have determined that TAK1 is required for proper differentiation of myeloid lineage and survival of macrophage precursors and/or mature macrophages. However, it is not clear whether TAK1 is important for maintenance of progenitors or mature macrophages and what the role of TAK1 is in activation of macrophages. Here we investigate the role of TAK1 and its binding partners, TAB1 and TAB2, in both mature naïve and activated macrophages, and have determined that, TAB1 and TAB2 are essential modulators of TAK1 activity and cell survival in LPS-activated macrophages.

## Materials and Methods

### Bone marrow cell isolation and macrophage differentiation

*Tak1^flox/flox^*, *Tab1^flox/flox^*, and *Tab2^flox/flox^* C57BL/6 mice were described previously [15], [22], [35]. *Rosa26.CreERT* and *Tnfr^-/-^* mice were purchased from Jackson Laboratories, and bred in our lab to produce the indicated genotypes [36]–[38]. Experiments performed *in vitro* required isolating bone marrow cells from *Tak1*, *Tab1* and *Tab2* mutant mice with *flox/flox* (WT), *flox/+ Rosa26.CreERT* (F+Cre) or *flox/flox Rosa26.CreERT* (iKO). Bone marrow derived macrophages (BMDMs) were generated by the standard procedure culturing bone marrow cells in 30% L929 cell-conditioned medium. To achieve gene deletion, cells were treated with 0.3 µM 4-hydroxytamoxifen (4-OHT) for 4 days. All animal experiments including *in vivo* LPS treatment described later were conducted with the approval of the North Carolina State University Institutional Animal Care and Use Committee (IACUC protocol # 11-138B). All efforts were made to minimize animal suffering.

### Crystal violet assay

BMDMs were plated onto 12-well plates at a concentration of 2×10^5^ cells per well and treated with 0.3 µM 4-OHT. In some experiments, BMDMs were subsequently treated with LPS (1 µg/ml) for 1 and 3 days. Cells were fixed using 10% formalin, and stained with 0.1% crystal violet. The dye was eluted and analyzed at 595 nm.

### Reagents and antibodies

Specific monoclonal and polyclonal antibodies against the following antigens were used: CD3ε (145-2C11), CD11b (M1/70), and B220 (RA3-6B2) (eBioscience); F4/80 (BM8)(BioLegend), phospho-TAK1, TAK1, TAB1, and TAB2 described previously; phospho-IκB, phospho-p38, IκB, and p38 (Cell Signaling). Necrostatin-1 (Nec-1) was purchased from Santa Cruz and applied to the culture at the final concentration of 50 µM. LPS is derived from source strain *Salmonella minnesota* ATCC 9700 (Sigma-Aldrich, catalog number L6261).

### Flow cytometry

BMDMs were detached from culture dishes and incubated with annexin V-Pacific Blue (BioLegend) and Fixable Viability Dye eFluor 780 (eBiosicence) for cell death analysis. Stained cells were analyzed on flow cytometer (BD Biosciences LSR II), and data were analyzed using FlowJo software (Tree Star). Events were gated to exclude debris based on forward scatter (FSC) and side scatter (SSC) profile, then gated on Pacific Blue (annexin V) or APC-Cy7 (fixable viability dye) when compared to unstained control.

### Western blotting

BMDMs were lysed in extraction buffer (20 mM HEPES [pH 7.4], 150 mM NaCl, 12.5 mM β-glycerophosphate, 1.5 mM MgCl_2_, 2 mM EGTA, 10 mM NaF, 2 mM DTT, 1 mM Na_3_VO_4_, 1 mM PMSF, 20 µM aprotinin, 0.5% Triton X-100) in ice for 15 minutes. Cells and debris were then pelleted by centrifugation at 20,000 G for 10 at 4°C. Cell extracts were resolved on SDS-PAGE and transferred to Hybond-P membranes (GE Healthcare). The membranes were immunoblotted with various antibodies, and the bound antibodies were visualized with horseradish peroxidase-conjugated antibodies against rabbit or mouse IgG using the ECL Western blotting system (GE Healthcare).

### Mouse model (in vivo)

For *in vivo* experiments, deletion of *Tab1* was achieved in *Tab1^iKO^* mice by intraperitoneal injection of 50 mg per kg tamoxifen on 3 consecutive days. After a period of 3-5 weeks, to reduce effects of Cre toxicity, whole blood was isolated and gene deletion was verified by Western Blot. To exclude the effect of Cre toxicity, we included F+Cre (*Rosa26.CreERT2 Tab1^flox/+^*) mice as controls. Mice were intraperitoneally injected with 8 mg/kg LPS, and were euthanized at 72 hours and dissected. Peritoneal leukocytes were collected by peritoneal lavage and collected in phosphate buffered saline (PBS), and splenocytes were harvested and prepared in a single cell suspension. Red blood cells were lysed by suspending cells in 0.83% NH_4_Cl lysis buffer and washed once with PBS. Cells were incubated with anti-CD16/32 in ice for 20 minutes to block FcγRII/III, followed by incubation with fluorophore-conjugated monoclonal antibodies (CD11b, B220 CD3ε, and F4/80) to evaluate cell type. Cells were washed in PBS and characterized on a BD LSRII flow cytometer (BD Biosciences). Data analysis was performed using FlowJo software (Tree Star).

### Statistical analysis

Cell counts were normalized to control and compared using a two-tailed Student's t-test. Values shown are means ± standard deviation with results considered significant if a probability of Type I error was <.05.

## Results

### Deletion of Tak1 or double deletion of Tab1 or Tab2 spontaneously kills bone marrow derived macrophages

**Figure 1:**
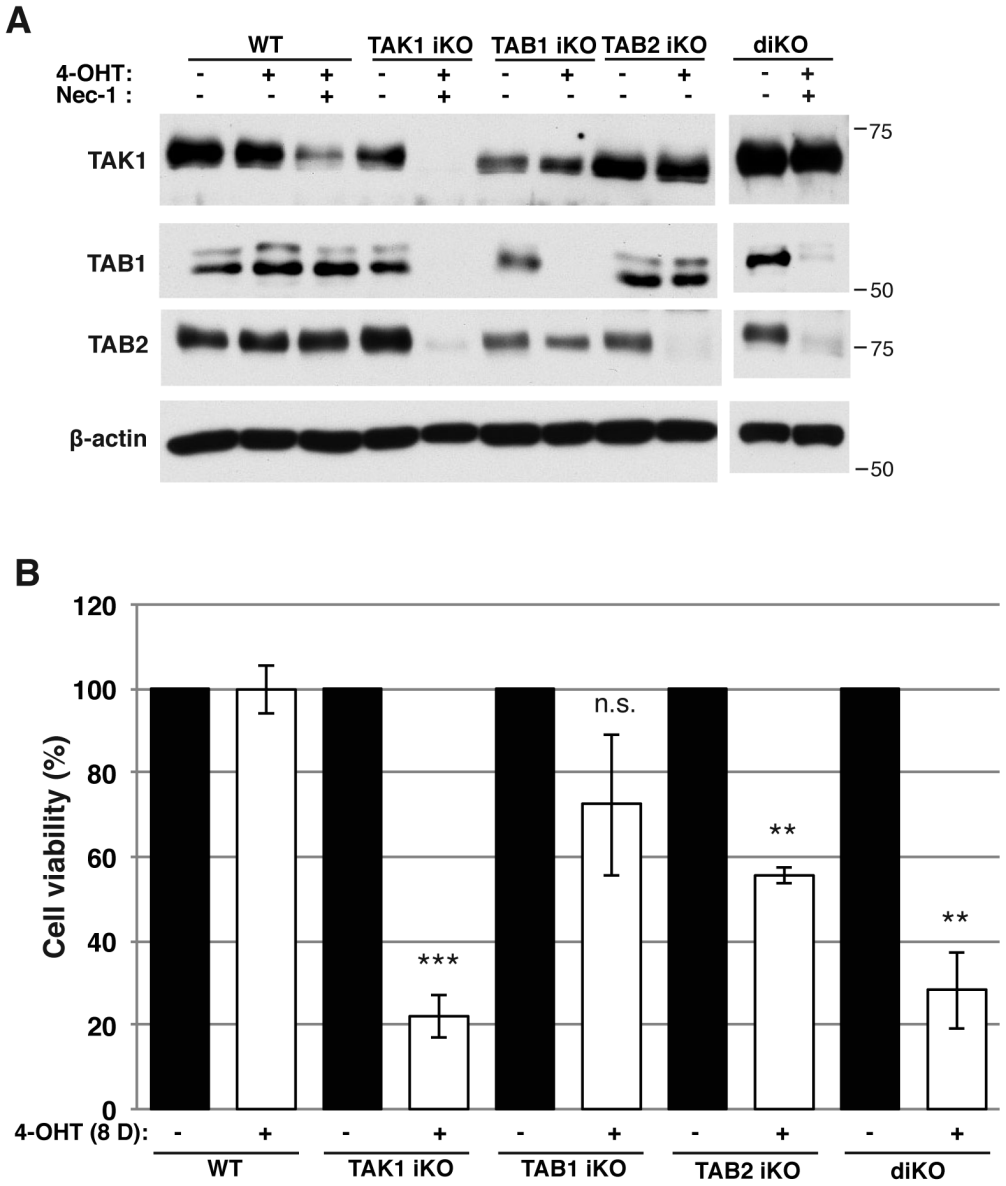
TAK1 is required for macrophage survival. (A) Western blotting analysis of TAK1, TAB1 and TAB2 in control, *Tak1^iKO^, Tab1^iKO^, Tab2^iKO^* and *diKO* BMDMs. Bone marrow cells were cultured in macrophage medium and treated with 0.3 µM 4-OHT or vehicle (ethanol) for 4 days. *Tak1^iKO^* and *diKO* BMDMs were additionally treated with 50 µM Necrostatin-1 (Nec-1). Anti-β-actin Western blotting was used as a loading control. The numbers beside each panel denote the size and the position of molecular weight markers. (B) Viability of WT, *Tak1^iKO^, Tab1^iKO^, Tab2^iKO^*, and inducible double-deficient *(diKO)* BMDMs. Cells were cultured for 8 days with 0.3 µM 4-OHT and stained with 0.1% Crystal Violet. Data are mean percentages of attached macrophages compared to ethanol-treated +/− SD for 3 independent experiments. Asterisks indicate p-values: **  =  P<0.005; ***  =  p<0.0005.

We characterized bone marrow derived macrophages (BMDMs) in adult mice having deletions of *Tak1*, *Tab1* or *Tab2* single or *Tab1* and *Tab2* double gene using the ubiquitously-expressed inducible Cre recombinase system, *Rosa26.CreERT* deleter mice. *Rosa26.CreERT Tak1^flox/flox^* (referred to as *Tak1^iKO^*), *Rosa26.CreERT Tab1^flox/flox^* (referred to as *Tab1^iKO^*), *Rosa26.CreERT Tab2^flox/flox^* (referred to as *Tab2^iKO^*), and *Rosa26.CreERT Tab1^flox/flox^ Tab2^flox/flox^* (referred to as *d^iKO^*) were compared with littermate or age matched controls, no-Cre *flox/flox* (referred to as WT or control). In some experiments, mice having heterozygous gene deletion, *Rosa26.CreERT flox/+* were also used as a control, which did not show any abnormality nor did WT. Bone marrow cells were differentiated to macrophages and gene deletion was subsequently induced by treatment of 4-hydroxytamoxifen (4-OHT) in macrophage culture medium, and vehicle (ethanol) was treated as control. The amounts of TAK1, TAB1 and TAB2 proteins were determined by immunoblotting at 4 days with 4-OHT or vehicle treatment. For *Tak1^iKO^* and *d^iKO^* macrophages, we additionally treated with necrostatin-1 (Nec-1), an inhibitor of receptor interacting protein 1 (RIP1), which is known to block *Tak1*-deficient macrophage death [34]. Nec-1 blocked cell death and allowed us to recover sufficient protein for Western blotting. We observed fluctuations in the amount of proteins among different litters and with Nec-1 treatment. Nonetheless, TAK1, TAB1, and TAB2 were greatly reduced within 4 days with 4-OHT treatment in *Tak1^iKO^* macrophages (Figure 1A). Reduction of TAB1 and TAB2 in *Tak1^iKO^* macrophages is presumably due to destabilization of unbound TAB1 and TAB2. In contrast, TAB1 or TAB2 but not TAK1 was reduced in *Tab1^iKO^* or *Tab2^iKO^* macrophages, respectively. Both TAB1 and TAB2 but not TAK1 were reduced in *d^iKO^* macrophages. These indicate that TAK1 is intact in *Tab1^iKO^*, *Tab2^iKO^*, and *d^iKO^* macrophages, which allow us to investigate the specific roles of TAB1 and TAB2 without altering the protein level of TAK1 in these macrophages. The number of macrophages declined at 8 days with 4-OHT treatment in *Tak1^iKO^* macrophages, whereas the number of control macrophages, including WT with 4-OHT and *Tak1^iKO^* with vehicle treatment, were not altered during the period of experiment (Figure 1B). Thus, TAK1 is essential for cultured macrophage integrity. These results are consistent with the previously reported results in myeloid-specific deletion of *Tak1* [34]. Thus, TAK1 seems to be important for survival of not only precursors but also mature naïve macrophages. In contrast to *Tak1* deletion, either *Tab1* or *Tab2* single deletion only moderately or slightly decreased the number of macrophages (Figure 1B). This indicates that TAB1 or TAB2 may partly contribute to but is not required for naïve macrophage survival. Importantly, we found that double deletion of *Tab1* and *Tab2* reduced the number of macrophages to a level similar to *Tak1* deletion (Figure 1B). Collectively, these results suggest that TAB1 and TAB2 may redundantly function to maintain TAK1 activity in naïve macrophages and TAK1 basal activity is required for survival of naïve macrophages.

### TAB1 and TAB2 are required for survival of LPS-stimulated macrophages

**Figure 2:**
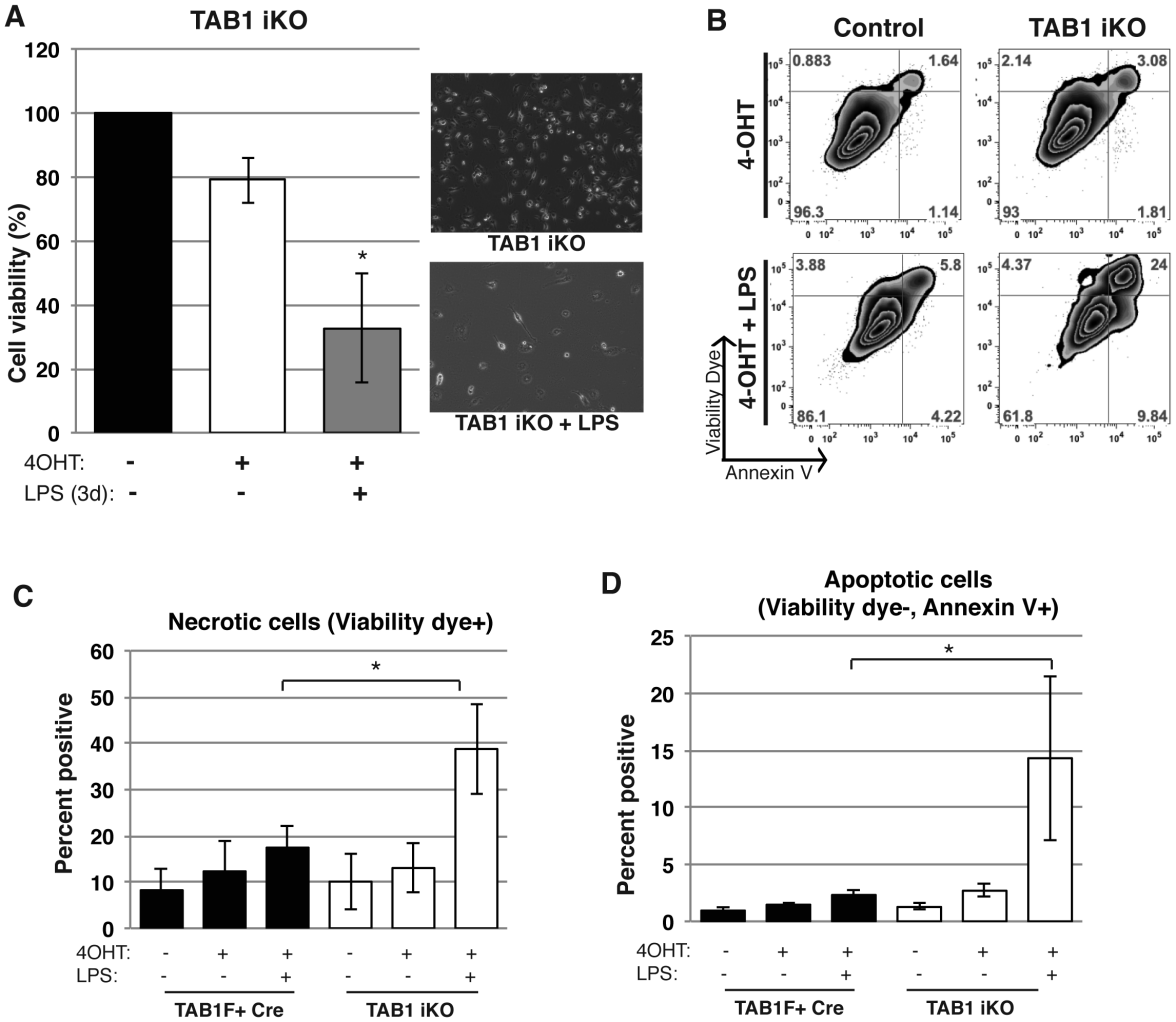
TAB1 is required for LPS-activated macrophage survival. (A) Viability of LPS-treated *Tab1^iKO^* macrophages. *Tab1^iKO^* and control BMDMs were cultured for 8 days with 0.3 µM 4-OHT followed by 3 days 1 µg/ml LPS. Viability was measured by Crystal Violet Assay, and data shown are mean percentages of attached macrophages compared to 8 days treated with vehicle +/− SD of 3 independent experiments. (B) Flow cytometry analysis of *Tab1^iKO^* BMDMs. *Tab1^iKO^* or *Tab1^F+^ Cre* BMDMs were cultured in macrophage medium with 0.3 µM 4-OHT or vehicle (ethanol) for 4 days. All cells including attached and floating cells were collected and stained with annexin V-Pacific Blue and Fixable viability dye eFlour 780, then analyzed on flow cytometer. Events were gated to exclude dead cells and debris, then gated on events positive for annexin V and fixable viability dye compared with unstained controls. Shown is representative figure of 3 independent experiments. (C and D) *Tab1^iKO^* and controls including WT and F+Cre BMDMs were cultured 3 days in 0.3 µM 4-OHT-containing macrophage medium, then 4 days with the addition of 1 µg/ml LPS. (C) Viability Dye-positive cells as a percentage of total cells is shown. (D) Graph shows Annexin V-positive but viability dye-negative cells. Graph represents results of four independent experiments +/− SD.

**Figure 3:**
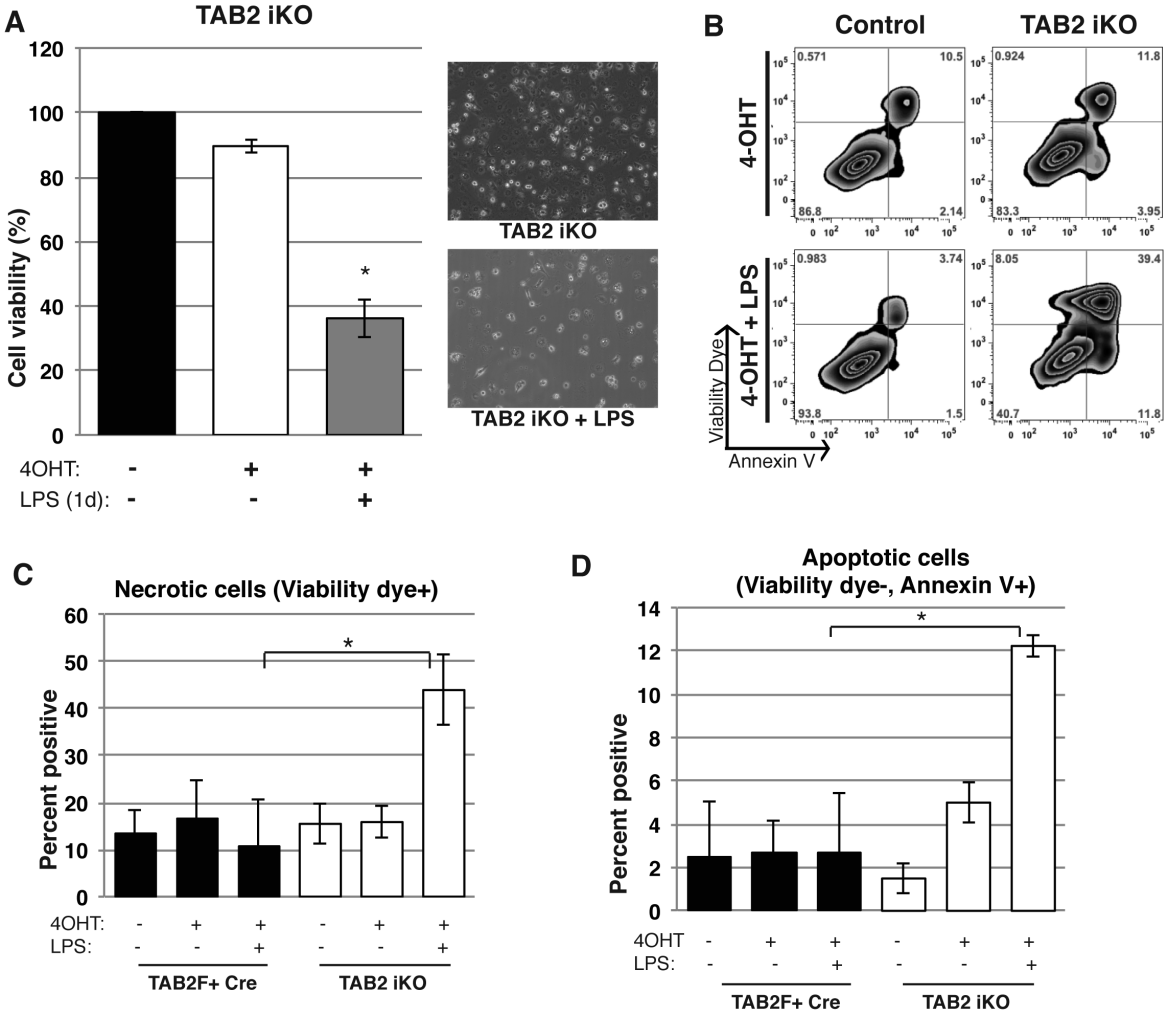
TAB2 is required for LPS-activated macrophage survival. (A) Viability of LPS-activated *Tab2*-deficient macrophages. *Tab2^iKO^* BMDMs were cultured for 8 days with 0.3 µM 4-OHT, then 1 µg/ml LPS was added to culture medium for 1 day. Data are mean percentages of attached macrophages compared to 8 days treated with 4-OHT alone +/− SD between 3 independent experiments as measured by Crystal Violet Assay. (B) Percentages of annexin V and viability dye positive cells. *Tab2^iKO^* and control (WT) BMDMs were cultured with 0.3 µM 4-OHT for 3 days then with 1 µg/ml LPS for 1 additional day in 3 independent experiments. Cells were stained with viability dye and annexin V and analyzed by flow cytometry. Percentages of single positive and double positive cells based on unstained controls in a representative experiment are shown. (C and D) Necrotic and apoptotic LPS-activated macrophages with *Tab2* deficiency. *Tab2^iKO^*, and controls including WT and F+Cre BMDMs were cultured 3 days in 0.3 µM 4-OHT-containing macrophage medium, then 1 day in medium containing 0.3 µM 4-OHT and 1 µg/ml LPS. (C) Necrotic cells are shown as percentage positive for viability dye. (D) Annexin V positive but viability dye-negative cells as a percentage of total cells is shown. Graphs indicate results of four independent experiments +/− SD.

To determine the role of TAK1 complex in activated macrophages, we treated *Tab1^iKO^ or Tab2^iKO^* macrophages with bacterial moiety, lipopolysaccharide (LPS). Wild type macrophages treated with LPS did not exhibit reduced cell viability in our experimental setting (Figure S1). Even in this condition, we found that activation of macrophages by LPS treatment noticeably reduced the numbers of *Tab1^iKO^* macrophages within 3 days (Figure 2A). *Tab1^iKO^* macrophages exhibited both increased annexin V binding and loss of plasma membrane integrity (Figures 2B). The percentage of cells showing a necrotic feature, cell permeability dye-positive, was greatly increased by LPS treatment (Figure 2C). Apoptotic cells characterized by annexin V binding-positive but permeability dye-negative were also increased by LPS treatments (Figure 2D). Thus, LPS-activated macrophages require TAB1 for their survival, and *Tab1* deficiency causes cell death having both necrotic and apoptotic features. Similarly, we found that *Tab2^iKO^* macrophages were significantly declined upon LPS treatment (Figure 3A). In contrast to *Tab1* deletion, *Tab2^iKO^* macrophage underwent cell death only one day after LPS treatment. LPS-treatment of *Tab2^iKO^* macrophages increased the frequency of annexin V-positive and cell permeability dye-positive population (Figure 3B). Similar to *Tab1^iKO^*, both necrotic and apoptotic cells were increased by LPS treatment in *Tab2^iKO^* macrophages (Figure 3C and 3D). These results demonstrate that, in contrast to naïve macrophages, both TAB1 and TAB2 are important for activated macrophage survival.

### LPS activates TAK1 through TAB1 and TAB2 in macrophages

**Figure 4:**
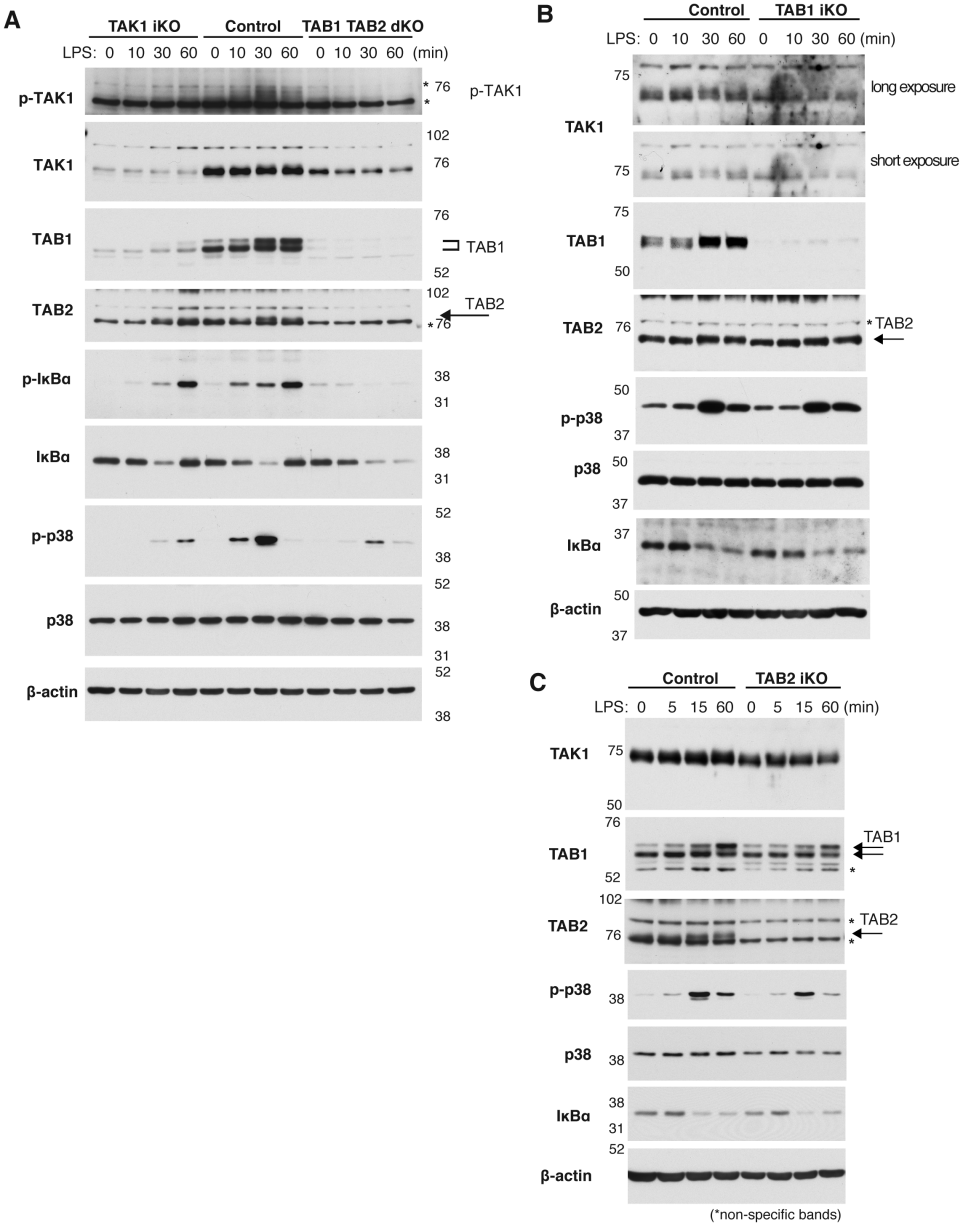
LPS activates TAK1 through TAB1 and TAB2. (A) Western blotting analysis of *Tak1^iKO^* and *Tab1Tab2^diKO^* and control BMDMs. Cells were cultured for 4 days with 0.3 µM 4-OHT in the presence of 50 µM Nec-1, followed by 1 µg/ml LPS treatment for the indicated period of time. Anti-βactin was used as a loading control. Asterisks indicate non-specific bands. (B) *Tab1^iKO^* or control BMDMs were cultured with 0.3 µM 4-OHT for 3 days then treated with 1 µg/ml LPS for the indicated period of time. Whole cell extracts were analyzed by Western blotting using the indicated antibodies. Asterisks indicate non-specific bands. (C) *Tab2^iKO^* or control BMDMs were cultured with 0.3 µM 4-OHT for 3 days then treated with 1 µg/ml LPS for the indicated period of time. Whole cell extracts were analyzed by Western blotting using the indicated antibodies. Asterisks indicate non-specific bands.

We hypothesize that TAB1 and TAB2 mediate LPS-induced TAK1 activation, which may be required for LPS-activated macrophage survival. To test this, we examined the levels of TAK1 activation and subsequent downstream events, activation of NF-κB and p38, following LPS stimulation. We note that, since *Tak1* or *Tab1* and *Tab2* double deletion spontaneously kills macrophages, we treated macrophages with Nec-1 to obtain live macrophages with *Tak1* or *Tab1* and *Tab2* double deletion. It is known that while RIP1 participates in NF-κB and p38 pathways, RIP1 catalytic activity is dispensable [39]. Consistent with this notion, NF-κB and p38 were activated in wild type macrophages even with Nec-1 treatment. We found that LPS activated TAK1 and its downstream IKK and p38 in macrophages (Figure 4A, middle 4 lanes), and *Tak1* deficiency reduced activation of both IKK and p38 (Figure 4A, left 4 lanes). TAK1 activity was monitored by phosphorylation of Thr 187 (Figure 4A, top panel), which is known to be associated with activation of TAK1 [40] However, non-specific bands were detected around the phosphorylated TAK1 in macrophages protein extracts, which were seen even in unstimulated macrophagse (Figure 4A, asterisks). Thus, we also utilized retardation of TAK1 band on SDS-PAGE to monitor TAK1 activation, which is caused by phosphorylation of several sites associated with TAK1 activation [41], [42]. While TAK1 exhibited migration shift upon LPS stimulation in wild type macrophages, *Tab1* and *Tab2* double deficiency abolished migration shift (Figure 4A, second panel), and reduced activation of IKK and p38 (Figure 4A, right 3 lanes) suggesting that LPS-induced activation of IKK and p38 is largely mediated by TAK1 and that TAB1 and TAB2 are essential for TAK1 activation in response to LPS. Deletion of either *Tab1* or *Tab2* impaired LPS-induced migration shift of TAK1, suggesting some impairment of TAK1 activation in response to LPS (Figures 4B and C, top panels). However, a single deletion of either *Tab1* or *Tab2* had a marginal effect on the LPS-induced degradation of IκB and phosphorylation and p38, suggesting that one of these proteins can activate TAK1 sufficiently at least in the pathways leading to activation of IKK and p38. These results collectively suggest that LPS-induced activation of TAK1 is mediated by TAB1 and TAB2, and that either deletion of *Tab1* or *Tab2* reduces TAK1 activation. However, LPS-induced activation of NF-κB and p38 seems to require at a minimum TAB1 or TAB2.

### LPS-induced cell death in Tab1- or Tab2-deficient macrophages is partially rescued by inhibition of RIP1

**Figure 5:**
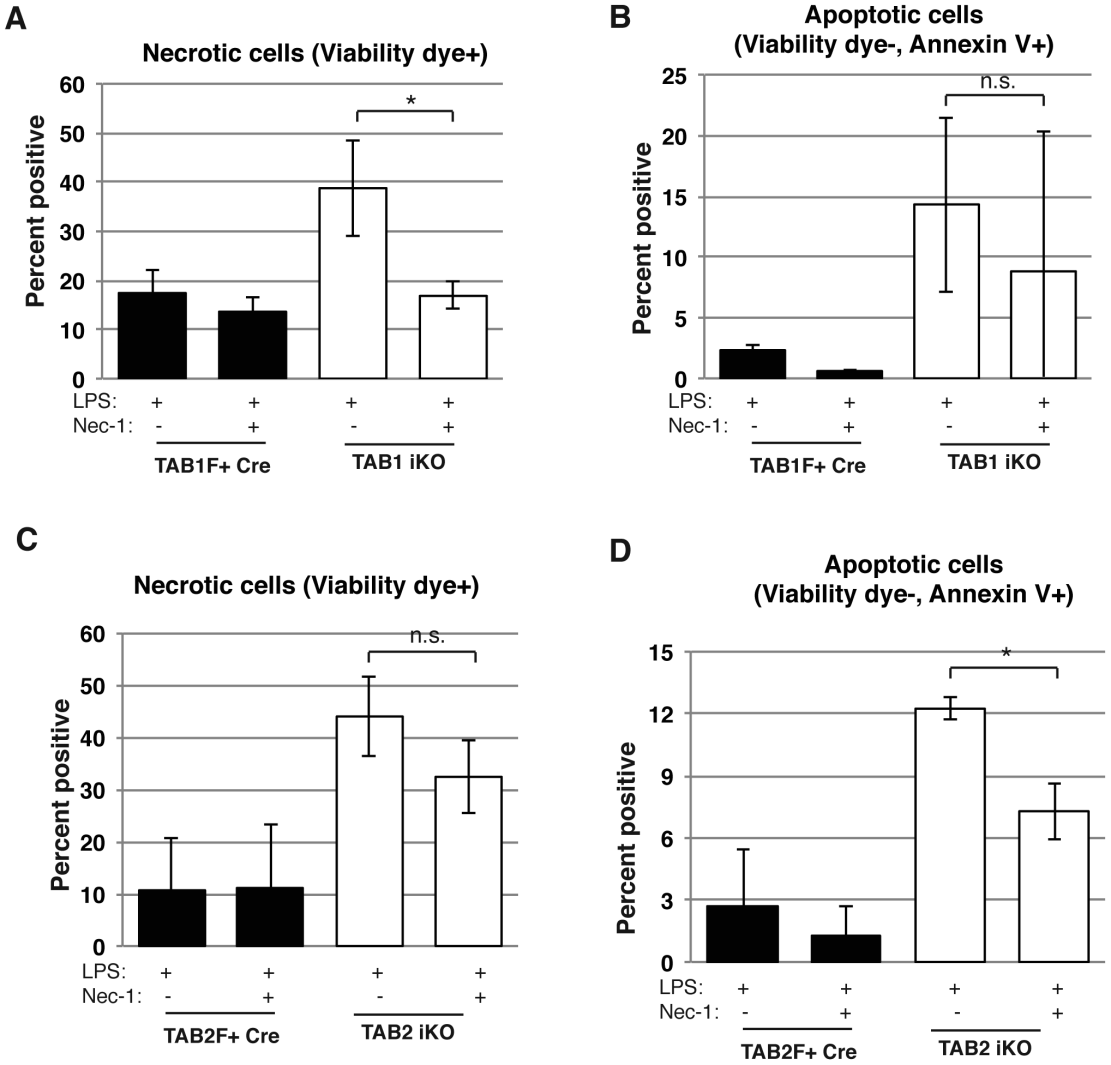
TAB1 or TAB2 deletion causes RIP1-dependent cell death. (A) *Tab1^iKO^* or control BMDMs were cultured with 0.3 µM 4-OHT with or without 50 µM Nec-1 for 8 days then treated with 1 µM LPS for 3 days, and viability was measured by Crystal Violet Assay. *  =  p<.05. (B) Viability of *Tab2^iKO^* BMDMs treated with RIP1 inhibitor. *Tab2^iKO^* BMDMs were treated with 0.3 µM 4-OHT for 8 days with our without 50 µM Necrostatin-1 (Nec-1), then treated with 1 µM LPS for one day. Viability was measured by Crystal Violet Assay. *  =  p<.05.

*Tak1* deletion is reported to cause RIP1-dependent cell death upon TNF treatment in several cell types [26], [27], [29]. Earlier study demonstrates that *Tak1*-deficient naïve macrophages die in a RIP1-dependent manner [34]. We hypothesize that activated macrophages die with the mechanism similar to that in naïve macrophages due to insufficient activity of TAK1. To test this, we examined the involvement of RIP1 kinase activity in *Tab1^iKO^* and *Tab2^iKO^* macrophage death by using Nec-1. In *Tab1^iKO^* macrophages, Nec-1 significantly and marginally reduced LPS-induced necrotic and apoptotic cells, respectively (Figure 5A and 5B). Nec-1 treatment exhibited some trends of reduction of cell death in *Tab2^iKO^* macrophages, although these did not reach to a statistic significance (Figure 5C and 5D). Both necrotic and apoptotic cells were decreased. These results suggest that RIP1 kinase activity may be at least partially involved in LPS-induced cell death in *Tab1^iKO^* and *Tab2^iKO^* macrophages.

### Tab1-deficient macrophages were reduced upon LPS injection in vivo

**Figure 6:**
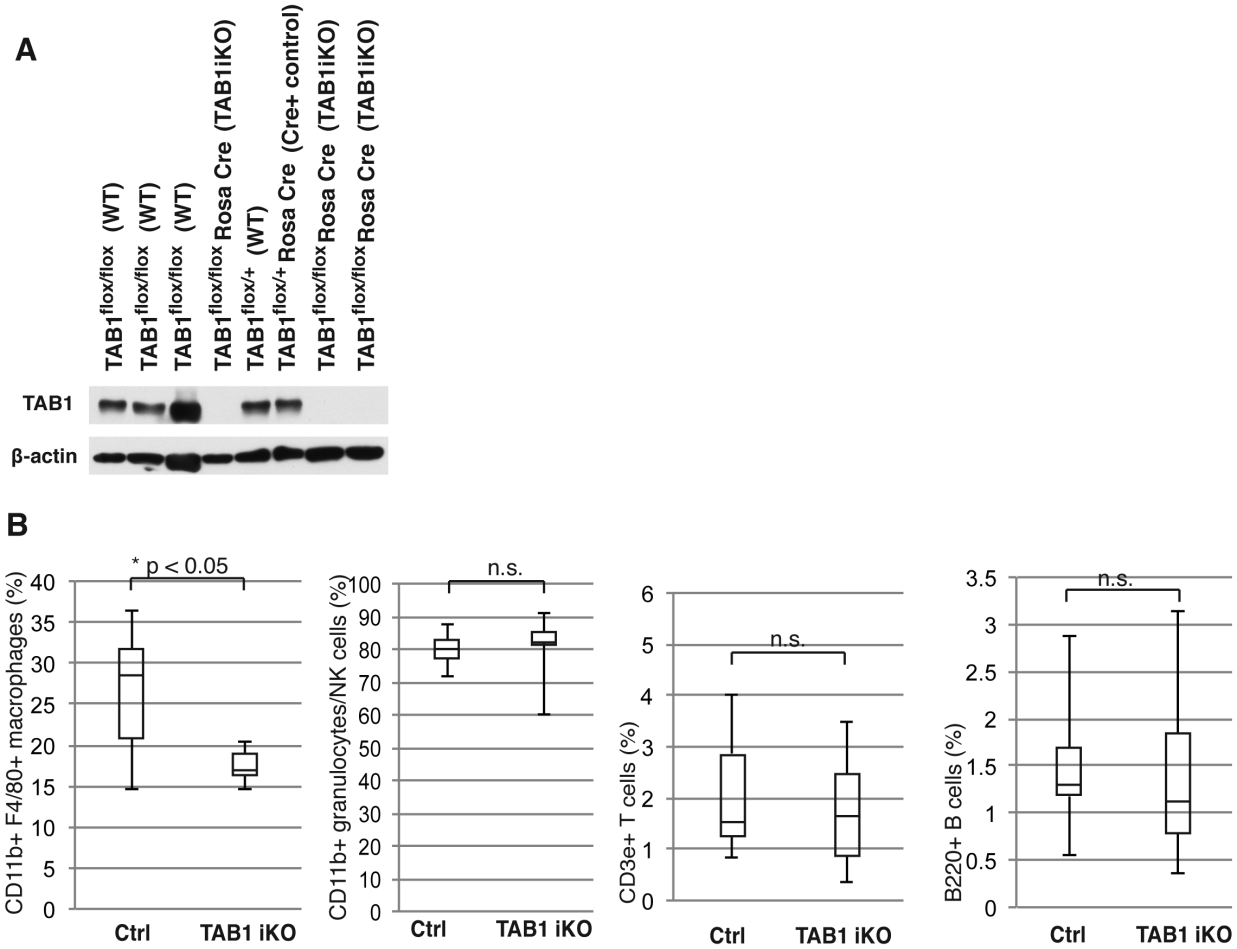
LPS treated *Tab1^iKO^* macrophages are reduced *in vivo*. (A) *Tab1^iKO^* or control including *Tab1^F+^* Cre mice were intraperitoneally injected with 50 mg/kg tamoxifen for 3 consecutive days. After 3–5 weeks, peripheral blood was collected and leukocyte extracts were tested for *Tab1* deletion by Western blotting. (B) *Tab1^iKO^* (n = 6) and control mice (n = 9) were intraperitoneally injected with 8 mg/kg LPS. Peritoneal leukocytes were collected at 72 hours and stained with fluorophore-conjugated antibodies. Shown is percent positive, excluding dead cells and debris, for the indicated markers. Percentages of CD11b+ F4/80+, CD11b+, CD3e+ or B220+ cells of total cells +/− SD is shown.

We next examined the role of TAK1 complex in macrophages *in vivo*. We anticipate LPS injection to recruit and activate monocytes from the bone marrow and circulation to the peritoneal cavity. Germline deletion of *Tak1* gene causes multiple defects in embryogenesis [43], [44], and deficiency of *Tak1* in adult mice also causes acute severe liver injury and mortality [25], [26]. We were not able to analyze *Tak1* deficient macrophages in adult mice due to these multiple acute effects. *Tab2^iKO^* mice did not exhibit overt abnormality upon gene deletion by tamoxifen injection; however, LPS stimulation caused acute liver dysfunction within 6 h (unpublished observations). *Tab2*-deficient macrophages were difficult to analyze in LPS-challenged mice for this reason. We then focused on *Tab1^iKO^* mice. *Tab1* gene was deleted in *Tab1^iKO^* mice by tamoxifen injection for 3 consecutive days. Although germline deletion of *Tab1* causes embryonic lethal phenotype [35], [45], we found that deletion of *Tab1* gene in adult mice did not cause overt abnormalities even though TAB1 proteins were greatly diminished (Figure 6A). To rule out the effect of Cre toxicity described previously [46], [47], the experiments were performed after more than 3 weeks post-tamoxifen injections. The number of peritoneal macrophages was determined in *Tab1*-deficient adult mice following LPS injection (Figure 6B). The frequency of CD11b+ F4/80+ macrophages in peritoneal fluid was lower in *Tab1^iKO^* mice compared to littermate controls following LPS stimulation. T-cells, B-cells and CD11b single positive cells were not significantly changed by *Tab1* deficiency. *Tab1* deficiency alone did not cause changes in macrophage population (Figure S2A). Importantly, the frequency of CD11b+ F4/80+ macrophages in peritoneal fluid was increased by LPS to around 30% from 10–15% under unstimulated conditions in control mice, but such increase was not observed in *Tab1^iKO^* mice. We noted that splenic CD11b+ F4/80+ macrophages were not significantly altered by LPS stimulation, and *Tab1* deletion did not cause any alteration of splenic macrophages (Figure S2B), supporting the notion that participation of TAB1-dependent pathway in macrophage survival varies depending on macrophage type or activation state [48]. These suggest that TAB1 is important for activated macrophage maintenance *in vivo*.

## Discussion

**Figure 7:**
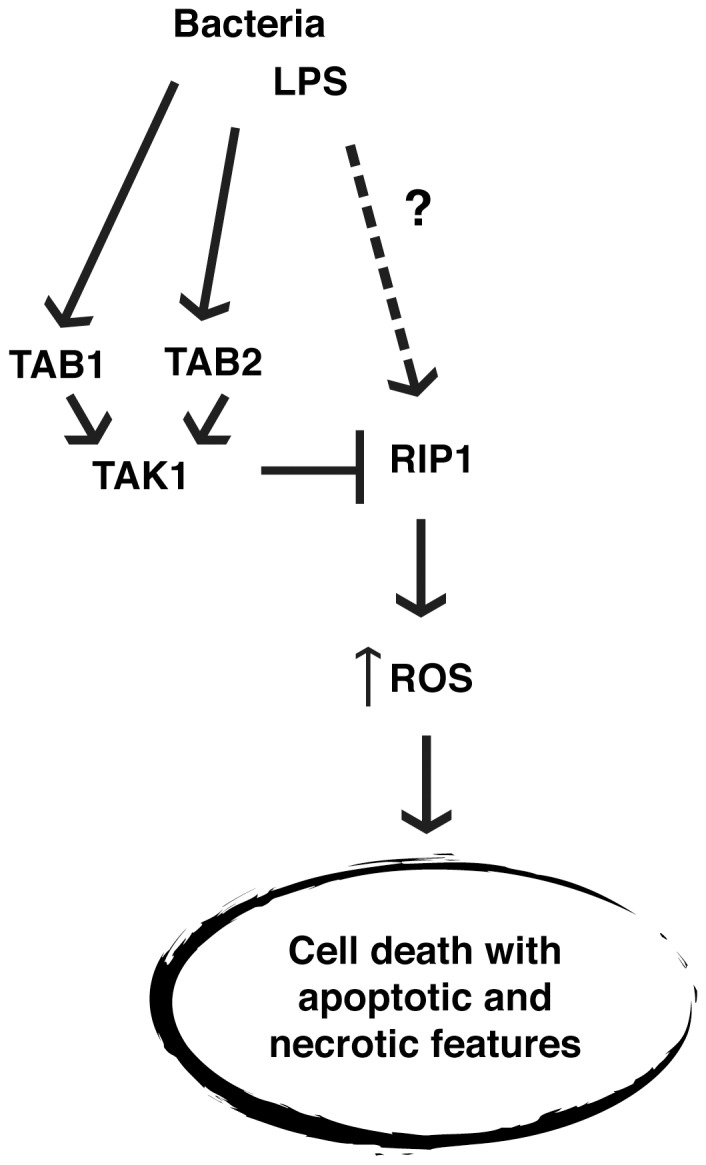
TAB1 and TAB2 are essential for LPS-activated macrophage survival. TAK1 binding proteins, TAB1 and TAB2 are essential for protecting BMDMs from LPS-induced cell death, which occurs downstream of RIP1, involves increased ROS, and shows features of both apoptosis and necrosis.

Our current studies identify an essential role for TAK1 modulator proteins, TAB1 and TAB2, in LPS-activated macrophages, in BMDMs, and in a mouse model of inflammation (Figure 7). The roles of TAB1 and TAB2 in macrophages have not been well characterized in the published literature to date. We demonstrate that TAB1 and TAB2 are essential for LPS-activated macrophage survival but are dispensable for survival of naïve macrophages. Bacterial moieties including LPS are strong activators of macrophages, which polarize macrophages toward an inflammatory, M1 phenotype. M1 macrophages mediate acute inflammation by secreting cytokines and chemokines, which play a major role in initiation of inflammatory responses [49]. These classically activated macrophages are prone to cell death, which contributes to the termination of inflammatory responses [3]–[5]. Chronically activated macrophages are associated with many disease conditions including obesity, autoimmune diseases, atherosclerosis and asthma [50]. In contrast, naïve macrophages in tissues, so-called resident macrophages such as microglia, Kupffer cells and Langerhans cells, play indispensable roles during development and in tissue repair [51]–[53]. Resident unstimulated macrophages are also important for prompt responses to protect tissues from insults. Therefore, the presence of unstimulated resident macrophages is beneficial to maintain tissue integrity. Thus, limiting inflammatory conditions by controlling only an activated sub-set of macrophages could be a useful tool in treating disease. Our results demonstrate that deletion of either *Tab1* or *Tab2* effectively kills only LPS-activated macrophages *in vitro*, and that *Tab1* deletion prevents increase of peritoneal macrophages upon LPS stimulation. These results indicate that TAB1 and TAB2 are potentially useful targets to selectively control the activated fraction of macrophages.

It is interesting that the *Tab1 Tab2^diKO^* shows diminished activation of IKK and p38 following LPS stimulation, which is similar to the *Tak1^iKO^* phenotype. When these genes are individually deleted, LPS-induced IKK and p38 activation are nearly normal and naïve macrophages persist. However, macrophages having deletion of either *Tab1* or *Tab2* could not withstand LPS-activation and *Tab1^iKO^* macrophages underwent necrosis after 3–4 days, while *Tab2^iKO^* die after 1 day, despite showing early LPS-induced TAK1 activation at or near WT levels (Figures 4B and C). In light of the nearly normal activation of NF-κB and p38 in *Tab1^iKO^* and *Tab2^iKO^* in response to LPS treatment, TAB1- and TAB2-dependent activated macrophage survival may occur through signaling pathway(s) independently of NF-κB and p38, which is in contrast to previous studies [4], [5].

Recapitulating our *in vitro* results, we found that the number of peritoneal macrophages was lesser in LPS-treated *Tab1^iKO^* mice when compared to control. Based on our *in vitro* data, we anticipate this disease model to produce a net increase in LPS-activated peritoneal macrophages in control but not *Tab1^iKO^* mice, however it cannot be conclusively excluded that the observed reduction is due to a defect in recruitment or expansion independent of cell death. Importantly, other hematopoietic cell types, including T cells, B cells and granulocytes, were found to be unaffected, suggesting TAB1-dependent survival signaling that is potentially unique to macrophages. We note here that liver was found to be undamaged in *Tab1*-deficient mice upon LPS injection under our experimental conditions, although higher doses of LPS are known to cause liver damage. Inferences based on our studies are limited by our mouse model, which has *Tab1* deleted in all cells, such that one cannot rule out the effects from other cell types *in vivo*. Future studies analyzing macrophage-specific conditional knockout mice for *Tab1* and *Tab2* could bring insight to the roles of these genes in macrophage cell death. Further research focused on this important mechanism in macrophages could inform inflammatory disease models, particularly diseases in which microorganisms target macrophages. The data supporting that TAB1 is essential for macrophage survival in LPS-treated mice could be used to improve our understanding of the control of inflammation.

## Supporting Information

Figure S1 Wild-type macrophages treated with LPS do not have reduced viability under experimental conditions. Viability of LPS-treated control macrophages. *Tab2^flox/flox^* BMDMs were cultured for 8 days with 0.3 µM 4-OHT followed by 3 days 1 µg/ml LPS, and viability was measured by Crystal Violet Assay. Shown are mean percentages of attached macrophages compared to 8 days treated with vehicle +/− SD of 3 independently performed experiments.
(TIF)

Figure S2 TAB1-dependent survival depends on type of macrophage. (A) Peritoneal leukocytes were collected from *Tab1*-deficient mice treated with vehicle (PBS) at 72 hours and stained with fluorophore-conjugated antibodies. Shown is percent positive of 2 control and 3 *Tab1^iKO^* for the indicated markers. Percentages of CD11b+ F4/80+, CD11b+, CD3e+ or B220+ cells of total cells ±SD is shown. (B) *Tab1^iKO^* and control mice were intraperitoneally injected with 8 mg/kg LPS. Splenocytes were collected and stained with fluorophore-conjugated antibodies. Shown is percent positive of 6 control and 4 *Tab1^iKO^* for CD11b+ F4/80+, CD11b+, CD3e+ or B220+ as a percentage of total cells ±SD.
(TIF)
