# Supplementary figures and images for: Activated Macrophage Survival Is Coordinated by TAK1 Binding Proteins
Source: PLoS One. 2014 Apr 15;9(4):e94982. doi: 10.1371/journal.pone.0094982 (PMC3988229; doi:10.1371/journal.pone.0094982)

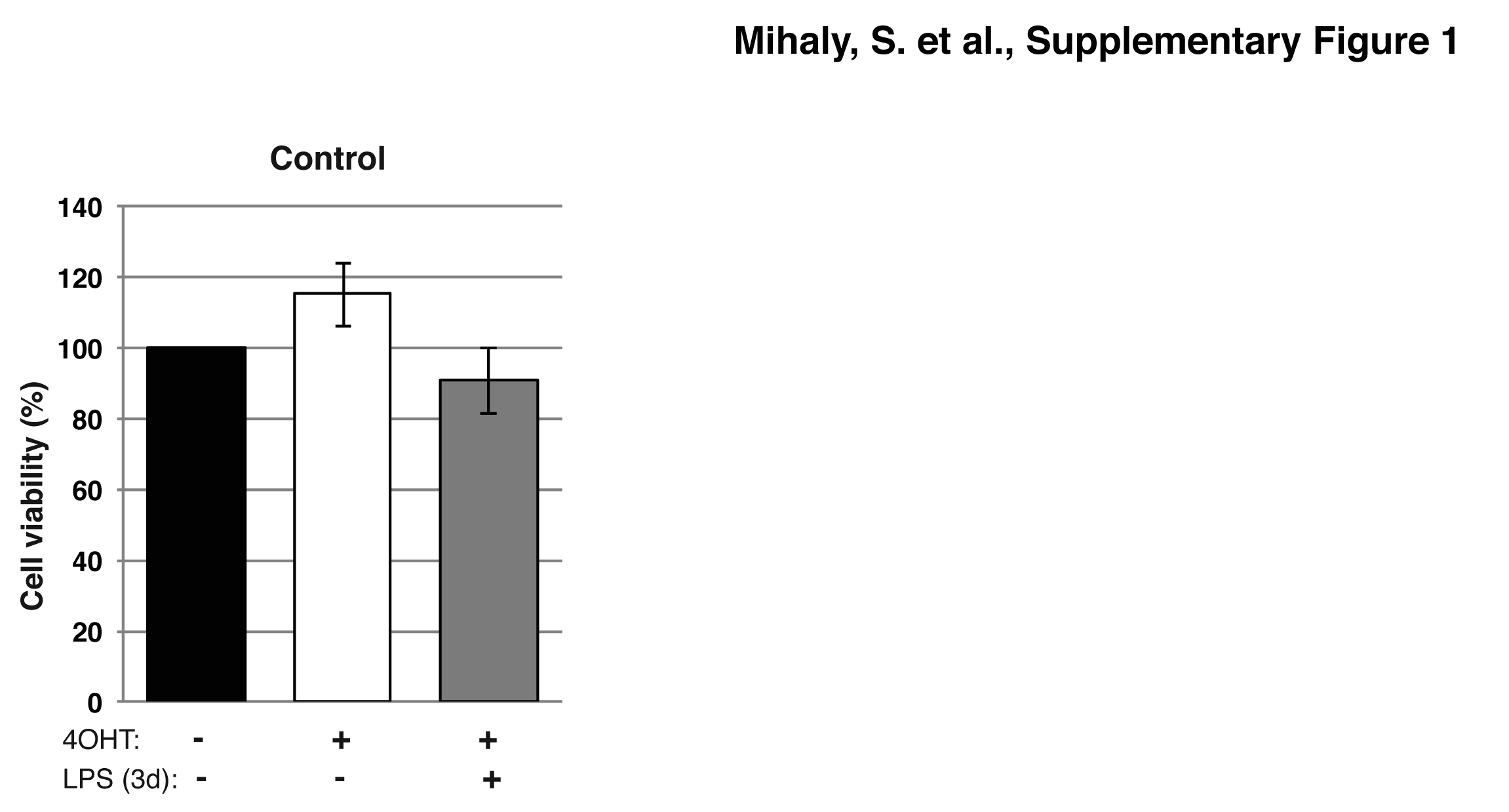

Supplement: Figure S1 — Wild-type macrophages treated with LPS do not have reduced viability under experimental conditions. Viability of LPS-treated control macrophages. Tab2flox/flox BMDMs were cultured for 8 days with 0.3 µM 4-OHT followed by 3 days 1 µg/ml LPS, and viability was measured by Crystal Violet Assay. Shown are mean percentages of attached macrophages compared to 8 days treated with vehicle +/− SD of 3 independently performed experiments. (TIF) [file pone.0094982.s001.tif]

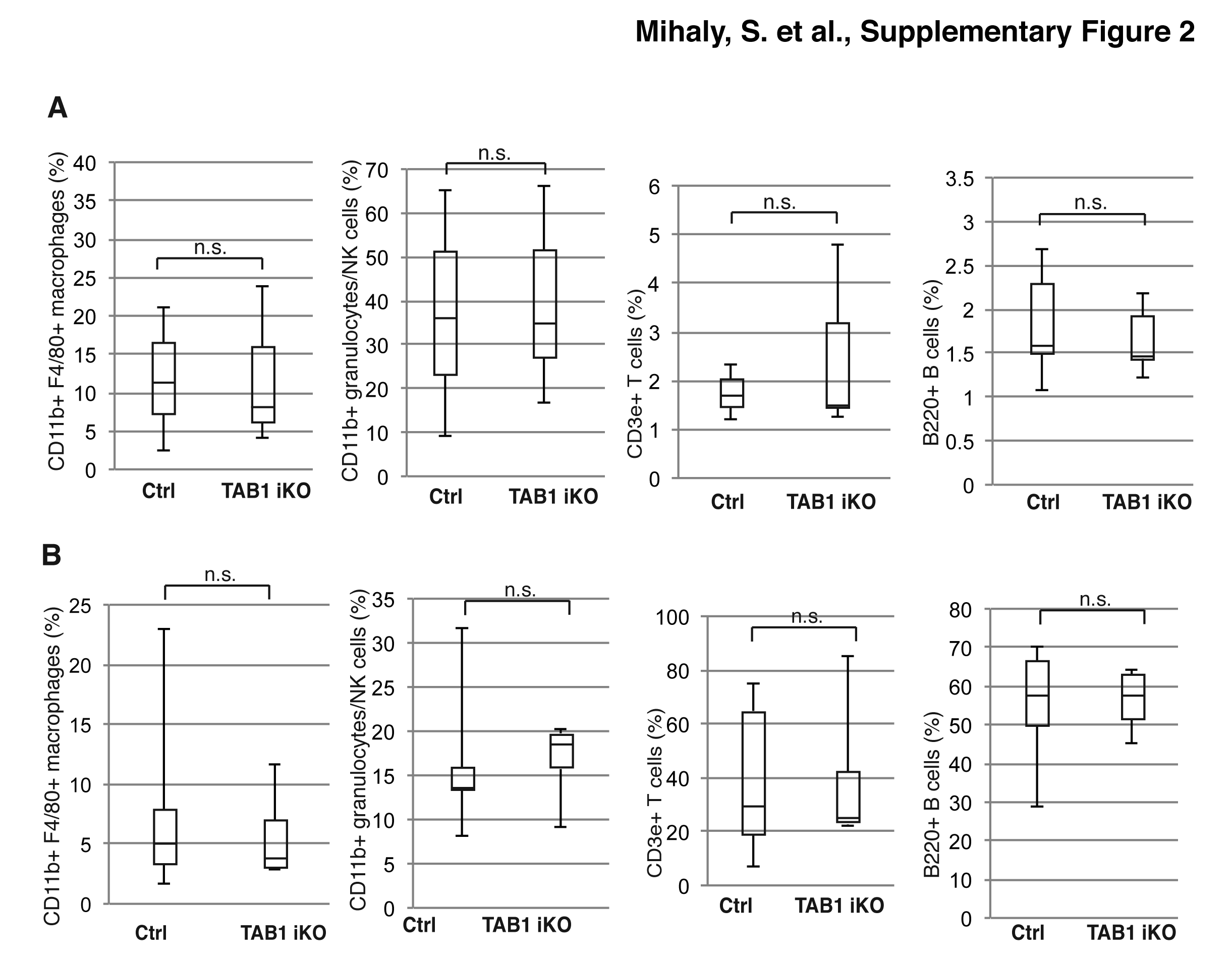

Supplement: Figure S2 — TAB1-dependent survival depends on type of macrophage. (A) Peritoneal leukocytes were collected from Tab1-deficient mice treated with vehicle (PBS) at 72 hours and stained with fluorophore-conjugated antibodies. Shown is percent positive of 2 control and 3 Tab1iKO for the indicated markers. Percentages of CD11b+ F4/80+, CD11b+, CD3e+ or B220+ cells of total cells ±SD is shown. (B) Tab1iKO and control mice were intraperitoneally injected with 8 mg/kg LPS. Splenocytes were collected and stained with fluorophore-conjugated antibodies. Shown is percent positive of 6 control and 4 Tab1iKO for CD11b+ F4/80+, CD11b+, CD3e+ or B220+ as a percentage of total cells ±SD. (TIF) [file pone.0094982.s002.tif]
